# Durable formulations of quorum quenching enzymes

**DOI:** 10.1038/s41598-025-12623-1

**Published:** 2025-07-28

**Authors:** Reed Jacobson, Colton Castonguay, Mikael H. Elias

**Affiliations:** 1 Department of Biochemistry, Molecular Biology and Biophysics, University of Minnesota, St. Paul, MN 55108 USA; 2 Biotechnology Institute, St. Paul, MN 55108 USA

**Keywords:** Biotechnology, Environmental biotechnology, Protein delivery

## Abstract

Enzymes with industrial potential often face limitations due to stability and longevity constraints. Thermostable quorum quenching lactonases are appealing biotechnology tools for controlling microbial pathogenicity and biofilm formation via the interference of quorum sensing. However, the effective formulation of these enzymes remains a challenge. Here, we evaluate the resistance and activity of two thermostable quorum quenching lactonase enzymes (SsoPox and GcL) across diverse formulations relevant to industrial applications. We systematically tested these enzymes with 16 different crop adjuvants (including oils, an anti-foaming agent, surfactants, deposition aids, a water conditioner, and a sticking agent) over a 210-day period, demonstrating broad compatibility except with oil-based adjuvants. Additionally, both enzymes maintained their activity when incorporated into five different coating bases (acrylic, silicone, polyurethane, epoxy, and latex) with activity levels varying according to polymer type. Further investigation of enzymatic acrylic coating characterized the effects of salt water and temperature on enzyme activity levels. Functionalized coatings maintained remarkable stability over 250 days in both wet and dry conditions. These findings establish a practical demonstration and framework for integrating quorum quenching lactonases into industrial materials and formulations, significantly advancing their potential for ‘real-world’ applications for microbial control across multiple sectors.

**Supplementary Information:**

The online version contains supplementary material available at 10.1038/s41598-025-12623-1.

## Introduction

Enzymes are promising environmentally friendly replacements for costly and polluting industrial processes and products. Enzymes are used across several fields including pharmacy, agriculture, food, and waste management^1^, and the global market for industrial enzymes is expected to reach an estimated $8.7 billion by 2030^2^. Although attractive targets due to their high selectivity and low environmental impact^3^, the use of enzymes in industry is limited by their properties. Most enzymes are unsuitable for use in industry as they have evolved in the context of cells, and as a result are not able to tolerate required chemicals, solvents, and temperatures, or simply do not have the longevity required for industrial processes^2,4–6^. Historically, most characterized enzymes, and consequentially most enzymes used in industry are derived from mesophilic organisms that grow under moderate temperatures (20–45 °C). For these reasons, producing enzyme-containing products or materials remains a challenge. There has been a surge in research in the last three decades on enzymes from extremophiles^7^, particularly those derived from thermophilic organisms that live at high temperatures^4,8,9^. Thermal stability is typically considered a desirable property for enzyme use in industry, as it often correlates with other advantages such as resistance to degradation from chemicals and proteases, allows for activity at high temperatures, and thermostable enzymes generally have a longer shelf-life^7,10^.

Here we focus on thermostable enzymes with the ability to control microbial behaviors via interference in microbial signaling. Indeed, numerous bacteria utilize Quorum Sensing (QS), a communication system based on small signaling molecules to coordinate a range of behaviors, most notably biofilm formation^11^. Lactonases are enzymes that can catalytically degrade signals used in QS (specifically *N-*acyl-homoserine lactones (AHL)) and thereby inhibit bacterial behaviors that are dependent on QS, such as virulence and biofilm formation^12–14^. The utilization of these enzymes to control microbial behavior is therefore promising across a number of application fields, including but not limited to crop disease prevention and antifouling coatings^15^. However, the lack of durable and active formulations hampers the use of this technology in microbial control.

Interference in microbial signaling is an attractive strategy to control virulence in crop disease. Plant microbial infections result in significant production losses^16^, and current viable treatments are limited, lacking^17^ or involve chemicals such as antibiotics that have been shown to contribute to the spread of resistance and off-target effects^16,18^. Numerous plant pathogens utilize quorum sensing, and specifically AHL-based QS, such as *Agrobacterium*^19^, *Dickyea*^20^, *Erwinia*^21^, *Pantoea*^22^, *Pectobacterium*^23^, and *Pseudomonas*^24^. This was leveraged in the treatment of crop diseases as several methods to control plant infection with QS inhibition have demonstrated viability, including interference in QS using transgenic *Amorphophallus konjac*^25^ to reduce soft rot infection, utilizing lactonase-expressing transformed *Pseudomonas fluorescens* to reduce symptoms of potato soft rot caused by *Pectobacterium carotovorum*^26^ (formerly *Erwinia carotovora*)^27^, or direct enzyme treatment using stabilized lactonase enzymes also reducing potato soft rot caused by *Pectobacterium carotovorum*^25^. However, these uses of lactonase enzyme suffer from various difficulties, such as stability of the genetically modified organism within the plant microbiome^28^, regulatory aspects pertaining to the use of such organism^28,29^, or the stability of the enzyme^30^. Efforts in the formulation of shelf-stable enzyme solutions that remain active over a long period of time are needed.

Along with reduction of plant disease, enzymes capable of interfering in QS can also be utilized to reduce biofilm formation^13,14^. Bacterial biofilm is a complex community of bacteria living attached to a surface encased in a matrix of EPS (extracellular polymeric substance)^31^, and these biofilms create a burden on numerous industries, and can form on medical devices leading to infections^32^. In marine environments, biofilm creates an anchoring substrate for other large organisms to adhere to, leading to biofouling^33^. These bacterial biofilms and macroorganisms can accumulate on vessels, and thereby reduce shipping efficiency, increase fuel usage, resulting in increased costs and increased greenhouse emissions^34^. Current methods to mitigate biofilms and biofouling mostly rely on the use of biocides^31,35,36^. Therefore, the use of a more environmentally-friendly solution such as enzymes to control biofilms is appealing^37^. A significant challenge with biological molecules such as enzymes resides in their low stability. Incorporating enzymes into coatings can be challenging because they need to remain active in polymers and solvents for long periods of time, preserve the mechanical properties of the coatings, and be active in seawater and at low temperatures^38,39^.

Overall, there is a need to advance enzyme formulation, including lactonases. Here, we evaluate the stability of two thermostable quorum quenching lactonases with a variety of commercially available products, including crop adjuvants and marine coating bases. Crop adjuvants are widely used to boost pesticide efficacy^40^, and antifouling additives are commonly added to marine coatings^41^, making it important to test quorum quenching lactonases in crop adjuvants and marine coatings to assess their potential use in industrially-relevant conditions. We chose one highly thermostable enzyme, SsoPox from *Saccharolobus solfataricus* (variant W263I, T_m_ = 87.8 °C)^42^ that was previously shown to resist a variety of denaturants^15^, be highly protease resistant^15^, and shown to operate in coatings and in the field^43–45^. We also selected the thermostable GcL isolated from *Parageobacillus caldoxylosilyticus* (T_m_ = 67.82 °C, Figure SI) for comparison. We question whether these enzymes can remain active in these different products, and whether these formulations can remain active over time. This work addresses an important gap in establishing active and durable formulations of lactonase enzymes that will serve as the foundation of ‘real-world’ microbial control evaluations, as well as contributes to defining our understanding of the resistance of some enzymes in chemicals.

## Materials and methods

### Enzyme production and purification

The mutant lactonase used in this study, SsoPox W263I, subsequently referred to as SsoPox in this work, was previously described^42^. SsoPox and WT-GcL were purified using previously described procedures^44^, adapted to a 75 L fermentation system (New Brunswick Scientific, NJ) by the University of Minnesota BioResource Center. Briefly, exponential phase cultures were induced at 23 °C with 0.2% arabinose, 0.2 mM CoCl_2_, and 0.25 mM IPTG (Isopropyl ß-D-1-thiogalactopyranoside) for 25 h. Cells lysis was performed in 20 mM Tris-HCl pH 8.0, 2 mM MgCl_2_ and using 11 KU of benzonase. Cells were subsequently centrifuged and supernatants were purified using a heat step (75 °C and 65 °C for 30 min for SsoPox W263I and GcL, respectively). Precipitated proteins were removed by centrifugation (15,000 g/30 min/4 °C) and supernatants were collected. Proteins were diafiltered and concentrated in 50 mM Tris-HCl pH 8.5 buffer and lyophilized (VirTis FreezeMobile 25 system; ATS Scientific Products).

### Enzyme tolerance to crop adjuvants

Lyophilized SsoPox and GcL were resuspended in *activity buffer* (50 mM HEPES, 150 mM NaCl, and 0.2 mM CoCl_2_ at pH 8) and prepared at 0.2 mg/mL (wt/vol). Six replicates of 1 mL SsoPox and GcL were prepared in separate 1.5 mL Eppendorf tubes and were left in activity buffer for 210 days at room temperature. Enzyme activity was assayed at days 0, 4, 7, 14, 21, 30, 60, 90, and 210. Enzyme activity was measured in a 96-well plate setup and a 200 µL reaction volume. In each well, 185 µL of activity buffer was added along with 5 µL of enzyme solution, and 10 µL of 1 mM paraoxon ethyl was used to start the reaction.

Enzyme activity in the presence of 16 crop adjuvants was evaluated using the lower concentration of the recommended range by the manufacturer of each product (Table S2). Adjuvants were classified into oils, de-foaming agent, surfactants, depositions aids, water conditioners, and sticking agents based on the manufacturer’s classification. When adjuvants were advertised as belonging to several categories and containing oils, they were classified as oils. Adjuvant ingredients identified from the product labels were grouped into plant-based oils, non-ionic surfactants, ionic surfactants, polymers, organic acids, salts, and petroleum products (Table S1**)**. To assess the relative activity (RA; Table 1) of the enzymes in each adjuvant, the average activity of the first three timepoints (days 0, 4, and 7) was normalized to the activity of the corresponding timepoints days 0, 4, and 7 of the control measurement of the enzyme activity in activity buffer.

**Table 1:**
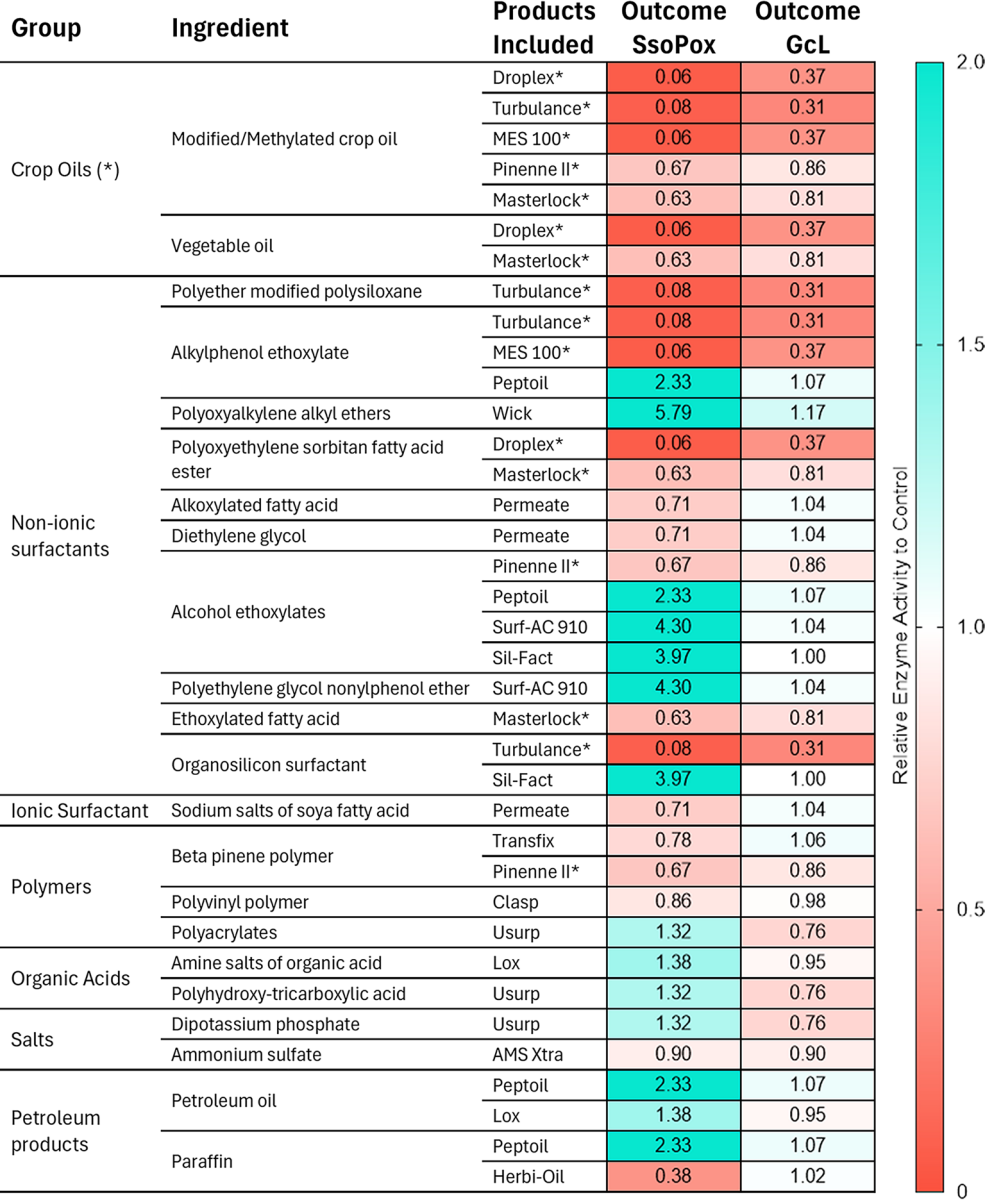
Effects of adjuvants on lactonase enzyme activity.

Ingredients from the crop adjuvants were collected from manufacturer labels and grouped into seven different categories: *Plant-based oils*, *non-ionic surfactants*, *ionic surfactants*, *polymers*, *organic acids*, *salts*, and *petroleum products*. Key ingredients are outlined in the “ingredient” column, and commercial adjuvant that contains them are noted. Products can appear multiple times if they contain multiple key ingredients. The relative activity (RA) levels for lactonases Ssopox and GcL are shown as a heat map where the activity from days 0, 4, and 7 were averaged, and normalized to control activity levels days (see “Enzyme tolerance to crop adjuvants” in methods). Adjuvants that contain plant-based oils are marked with an asterisk (*), and adjuvants that only contain a single ingredient are marked with a cross (^†^).

### Coating formulations and activity measurements 

Lactonase enzymes were evaluated in five different coating bases: Sher-Clear 1 K Waterborne Acrylic Clear Coat (acrylic), Elkem Bluesil ESA 7246 (silicone), Sherwin Williams - Acrolon 218 HS (polyurethane), Sherwin Williams Macropoxy 646 (epoxy), and Painters Touch Ultra Cover Premium (latex). For the acrylic coating, lyophilized SsoPox or GcL enzymes were dissolved in deionized water (DIW) to a concentration of 50 mg/mL, and subsequently diluted 1:50 in the acrylic coating to a final concentration of 1 mg/mL and mixed via inversion. For all other coatings, lyophilized enzymes were mixed directly into the paints at 1 mg/mL.

To apply the paint, 10 µL of the enzyme coating was distributed evenly into the well of a flat-bottom polystyrene 96-well plate (Fisher Scientific, USA). Any bubbles that formed in this process were popped. The painted plates were placed in a dark environment at room temperature uncovered for 24 h, unless otherwise specified.

Activity assay of the enzyme activity of the coatings was performed by adding 190 µL of *activity buffer* and 10 µL of 20 mM substrate ethyl-paraoxon (Sigma Aldrich, USA) (1 mM final) directly to coated microplate well (200 µL final). The enzyme activity quantification of the enzyme leakage in water was accomplished by adding 100 µL of water that the coating was submerged in to 90 µL of *activity buffer* and 10 µL of 20 mM ethyl-paraoxon (1 mM final) to a different microplate (200 µL final). Production of the paranitrophenolate anion was monitored at 412 nm for 90 min using a microplate spectrophotometer (Synergy HTX, BioTek, USA), as previously described^46^. This assay takes advantage of the ability of both SsoPox and GcL to hydrolyze this organophosphorus compound, the latter hydrolysis resulting in the production of the chromogenic anion paranitrophenolate. Enzymatic activity is reported as enzymatic units U, which is equivalent to 1 µM of substrate catalyzed per minute per mg of enzyme.

### Long term coating durability experiment

To evaluate the longevity of enzymatic coatings, 96-well plates were coated as described above, and half of the wells were filled up with 200 µL of DIW whereas the other half was left dry. Plates were sealed with microplate sealing tape (Thermo Scientific) and stored in the dark (to limit the possibility of photodegradation) at ambient temperature (23 °C).

Lactonase activity was measured at 1, 5, 7, 12, 14, 30, 45, 60, 90, 120, 150, and 240 days. Thiobutyl butyrolactone (Enamine, US) (TBBL; 1 mM final concentration) was used as a substrate as previously described^47^. Briefly, the bond between the sulfur group and the lactone ring of TBBL is cleaved creating a free sulfhydryl group. This subsequently reacts with DTNB (Ellman’s Reagent) (Alfa Aesar, USA) to create TNB, which produces a yellow color and can be read in a spectrophotometer at 412 nm^48^. Enzyme activity that leaked in the DIW in the submerged condition was assayed by sampling 100 µL, placed in a fresh 96 well plate along with 50 µL activity buffer, 40 µL Ellman’s Reagent and 1 mM TBBL. The reaction was monitored at 412 nm for 90 min. Enzymatic activity (U) is defined as 1 µM of substrate catalyzed per minute per mg.

### Glutaraldehyde enzyme immobilization

Lactonase crosslinking was performed with acrylic based coating. Prior to painting, glutaraldehyde (Millipore, USA) was added to each lactonase coating formulation (3% of total volume) and mixed via vortexing. 96-well plates were painted as described above and coatings were cured in the dark at room temperature and left to cure for 1 or 14 days. Enzymatic activity as reported by ethyl-paraoxon hydrolysis was monitored as described above.

### Evaluation of coating activity in different conditions

Coated 96-well plates were used to evaluate the effect of different conditions on the enzyme activity using acrylic based coating. The effect of salinity was evaluated by adding 200 µL of DIW or Reconstituted Sea Water (RSW; Instant Ocean, USA). Plates were then covered with Microplate sealing tape (Thermo Scientific, USA) to prevent evaporation, and stored for 24 h in the dark at room temperature. Activity using ethyl-paraoxon (1 mM final) was subsequently monitored by measuring absorbance at 412 nm for 90 min.

Similarly, the effect of temperature on the enzymatic coating activity was evaluated. Here, coated plates were incubated in the dark at 4 °C or ambient temperature (23 °C) for 3 h. Activity using ethyl-paraoxon (1mM final) was subsequently monitored by measuring absorbance at 412 nm for 90 min.

### Biofilm Inhibition assay

The bottom of the wells of 12-well plates (Corning, USA) were coated using 100 µL of enzyme/coating mixture and cured for 24 h in the dark at room temperature. A liquid culture (5 mL) of *Pseudomonas aeruginosa* PA01 was grown in LB medium overnight at 37 °C with 200 RPM shaking. The culture was diluted to an optical density of 0.2 and subsequently diluted 1:1000 in M63 Minimal Media (7.5 mM (NH_4_)_2_SO_4_, 50 mM KH_2_PO_4,_ 1.5 µM FeSO_4_·7H_2_O) with 0.2% glucose. Each coated well was filled with 2 mL of that inoculum. The plates were then sealed with a breathe-easy membrane (USA Scientific INC., USA) and incubated in the dark at 30 °C and shaking at 300 rpm for 20 h.

Biofilm growth quantification was performed as previously described^49^. Briefly, the wells were washed gently with 2 mL of DIW and allowed to air dry for 90 min at room temperature. 400 µL of 0.1% crystal violet dye in DIW was subsequently added and plates were incubated for 10 min. The wells were then washed with DIW three times to wash off unbound crystal violet, and were air-dried for 2 to 4 h at room temperature. Once dry, 1 mL of 33% Acetic Acid in DIW was added to each well to remove and solubilize the crystal violet dye for 10 min at ambient temperature. The dissolved dye was subsequently transferred into a new 96-well plate, and quantified by reading absorbance at 585 nm.

The data for this study is available at the Open Science Foundation (DOI 10.17605/OSF.IO/KJBY8).

## Results and discussion

### Formulation of lactonases in crop adjuvants

**Fig. 1:**
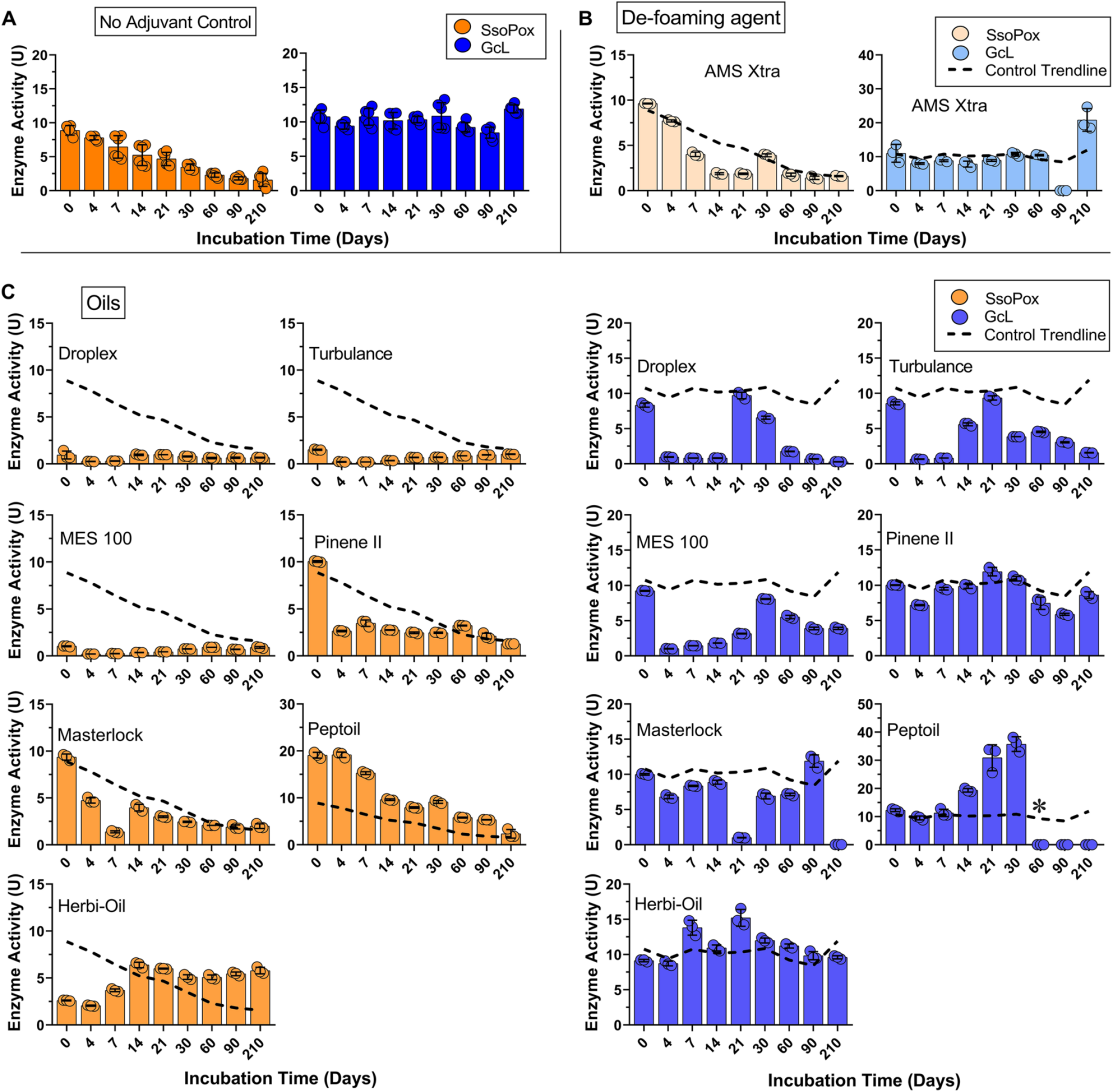
Lactonase enzymes show different levels of activity over time in different crop adjuvant classes – Group 1. (**A**) SsoPox (orange) and GcL (blue) enzymes were tested periodically for activity in activity buffer (no adjuvant control) over 210 days. This trace was used as the trendline (reference) for all subsequent graphs. Enzymes were also tested for activity in several classes of crop adjuvants including: (**B**) de-foaming agent, (**C**) oils (*indicates timepoint at which sample evaporated during experiment). The activity of lactonases SsoPox (orange) and GcL (blue) (0.2 mg/mL) were measured in the presence of manufacturer’s recommended concentrations of adjuvants days (see “Enzyme tolerance to crop adjuvants” in methods). Activity over time was tested via a paraoxonase assay. Enzyme activity is reported as U, which is 1 µM of substrate catalyzed per minute.

**Fig. 2:**
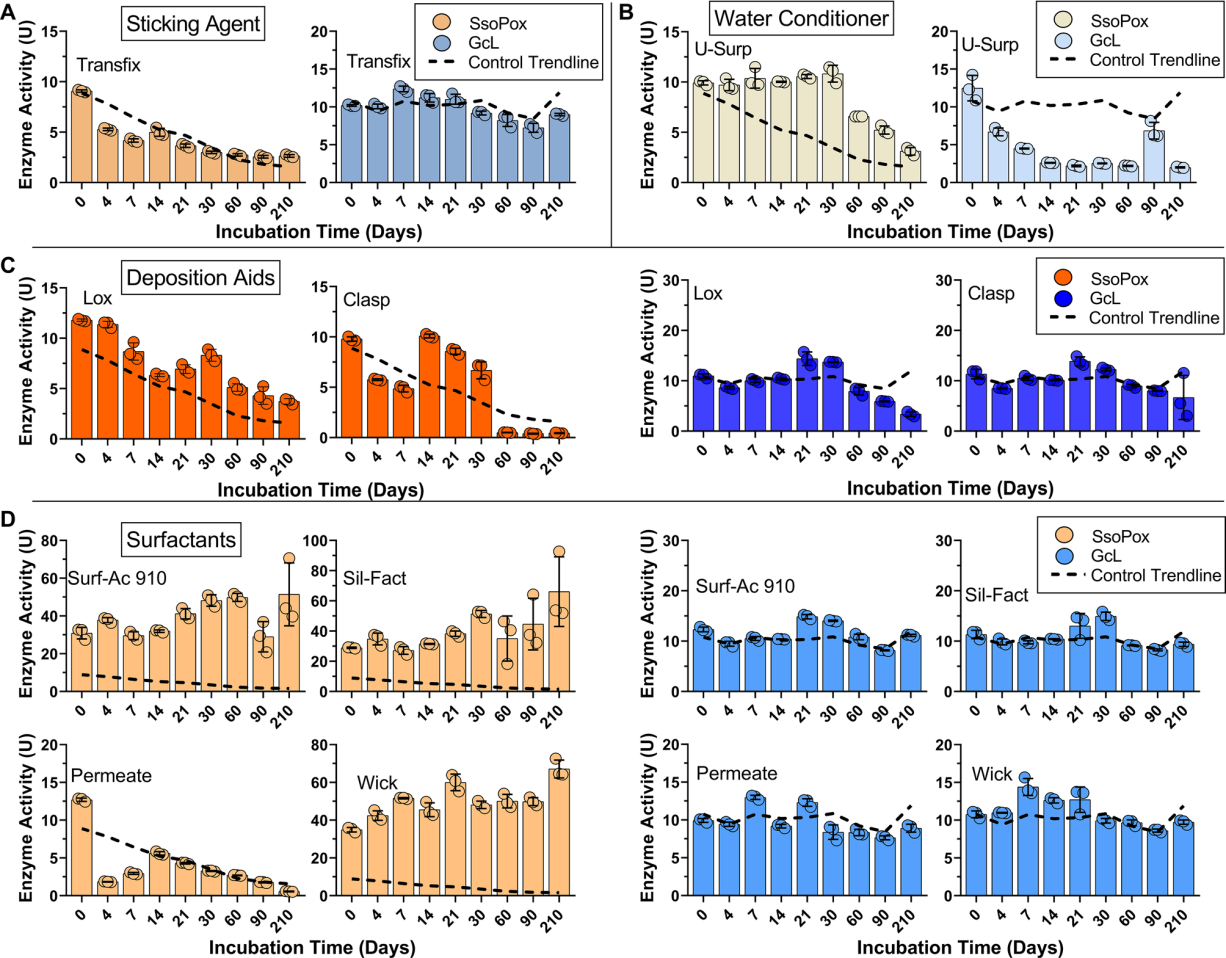
Lactonase enzymes show different levels of activity over time in different crop adjuvant classes – Group 2. Enzyme activity in presence of (**A**) surfactants, (**B**) deposition aids, (**C**) water conditioner, and (**D**) sticking agent. The dotted trendline (black) is the enzyme activity in the control conditions (activity buffer) and serves as reference. The activity of lactonases SsoPox (orange) and GcL (blue) (0.2 mg/mL) were measured in the presence of manufacturer’s recommended concentrations of adjuvants (see “Enzyme tolerance to crop adjuvants” in methods). Activity over time was tested via a paraoxonase assay. Enzyme activity is reported as U, which is 1 µM of substrate catalyzed per minute.

A simple way to functionalize enzymes is to use them directly in solution. Adjuvants are chemical additives that are used to improve pesticide performance in agriculture^50^. Because interference in QS has the potential to protect crop from infection and spoilage^51–53^, we evaluated the compatibility of two lactonases, SsoPox and GcL with 16 different crop adjuvants. The tested adjuvants were selected for their diverse chemical composition, and cover six different functional classes including *oils*, *anti-foaming agent*, *surfactants*, *deposition aids*, *water conditioner*, and *sticking agent* (Table S1). Testing the compatibility of enzymes in these chemicals will inform on the viability of potential use of lactonases in crop protection. Enzyme activity was reported in unit “U”, which is equivalent to 1 µM of substrate catalyzed per minute per mg of enzyme. To benchmark the activity levels of lactonases in these different productions, we evaluated the activity of the enzymes in *activity buffer* (see Methods) at ambient temperature over time (Fig. 1A). Over the time-course of the experiment (210 days), Ssopox lost ~ 83% of its initial activity (from 9 U to 1.5 U). This loss in activity is significantly larger than previously reported stability at room temperature in powder form (modest activity losses over 300 days^15^), and may be the result of hydrolysis in aqueous environment. On the other hand, GcL remained active over the tested period (10.5 U to 12 U). The stability over time of the enzyme preparation were subsequently used as trendlines to allow for simple comparison of the enzymatic activity in presence and absence of adjuvant (Figs. 1 and 2).

Examination of lactonase activity profiles suggest some general behaviors. First, it shows that one lactonase enzyme (SsoPox) shows much more activity variation with adjuvants than the other (GcL; Table 1). The *de-foaming agent* AMS X-tra shows very similar activity profiles compared to the control for both lactonases, suggesting that it has little to no effect on their activity levels over the tested period (Fig. 1B). The *oils* adjuvant category, that include Droplex, Turbulance, MES 100, Pinene II, Masterlock, Peptoil, and Herbi-Oil, were generally destabilizing for both enzymes (Fig. 1C). For example, the lactonase SsoPox shows low activity levels in Turbulence, Droplex, and MES 100 from day 0 (~ 85% reduction as compared to control), and the activity remains consistently low over the recorded 210 days. With Pinenne II and Masterlock, the initial activity at day 0 is similar to control, but the activity is lower than control in the first week by ~ 45% and ~ 78% respectively, and the overall profile over 210 days is similar to control (Fig. 1C). These observations are largely similar for lactonase GcL, for which activity in Turbulence, Droplex, and MES 100 dropped by ~ 90% in the first week. The activity of GcL remained constant (and similar to control) for all other tested oils for at least 30 days. GcL activity had transient increases at days 21–30 for some of the tested oils, and this potentially due to reversible changes in enzyme conformation leading to a re-gain and subsequent loss in activity in *oil* adjuvants.(Fig. 1C). It is not clear what specific ingredient causes the observed loss of activity. Turbulence, Droplex, MES 100, Pinenne II and Masterlock contain combinations of modified plant-based oil, methylated plant-based oil, or vegetable oils that might be the cause for activity loss. Peptoil and Herbi-oil do not contain plant-based oils, as instead they have petroleum-based oils. These adjuvants were overall better tolerated by the enzymes, particularly Herbi-oil that led to increased enzyme activity over the recorded time period of 210 days for both enzymes. This observation is consistent with previous work showing that denaturing agents can stimulate the enzymatic activity of SsoPox^15^. Overall, these results suggest that the tested enzymes (particularly SsoPox) are compatible with four out of the tested seven *oils* (Herbi-oil, Masterlock, Peptoil, Pinenne II) for long term usage.

The *sticking agent* Transfix shows very minimal effects on the enzymes’ activity levels (Fig. 2A). This is in contrast with the tested *water conditioner* U-surp, for which enzymes showed opposite behaviors. Indeed, the activity of SsoPox was globally preserved over the time course of the experiment, with all measured activity levels higher than control, whereas GcL showed significantly lower activity levels as compared to control (~ 75% reduction at day 14) (Fig. 2B). The two *deposition aids* that were tested, Lox and Clasp, had modest effects on the activity levels of the two lactonases over 210 days (Fig. 2C). SsoPox activity levels were higher over the monitored time period (e.g. ~ 30% higher than control at day 30), whereas GcL was mostly unaffected by Lox, except for the last time points (90, 210 days) where activity appears lower (Fig. 2C). With Clasp, the activity profiles of both enzymes are largely comparable to their relative controls, except for the three last time points for SsoPox (60, 90, 210 days) where the enzyme appears nearly inactive (Fig. 2C).

The last tested group, the *surfactants*, include Surf-Ac 910, Sil-Fact, Permeate and Wick (Fig. 2D). The activity levels of GcL remained largely stable over 210 days at levels similar to control with all four surfactants (Fig. 2D). For SsoPox, activity was mostly similar to control over the experiment for Permeate. However, the activity was largely stimulated with the three other surfactants (up to 6-fold the activity levels of day 0 with Wick at day 210; Fig. 2D). We observe that these compounds not only stimulated SsoPox activity levels, but also appear to reduce activity loss over time, with activity level profiles that are overall constant at high levels over 210 days. The stimulation of SsoPox activity is consistent with a previous report showing its activity stimulation in presence of the detergents sodium dodecyl sulfate (SDS) and sodium deoxycholate (DOC)^54–56^.

Overall, this work demonstrates that these two enzymes remain active for a substantial amount of time in conditions that are typically considered inappropriate for enzyme storage (ambient temperature and aqueous buffer) as well as in a range of adjuvants, some of which appear to stimulate and preserve their activity.

To further analyze these results, adjuvants were grouped based on their ingredients (Table 1). The analysis of the relative activity (RA; an activity level normalized to the activity of enzymes in activity buffer, see Methods) levels of both tested lactonases in the presence of adjuvants could reveal some preferences of the enzymes. However, this is complicated by the fact that numerous products contain compounds from several categories. Yet, results show that *Plant-based oils* appear to be mostly deleterious for the activity of both lactonases. For example, methylated/modified plant-based oils and vegetable oils had negative impacts on activity, and SsoPox was most negatively affected (RA = 0.06–0.63) while the activity of GcL was more modestly reduced (RA = 0.31–0.86).

Non-Ionic surfactants were overall well tolerated by the enzymes. Indeed, alkylphenol ethoxylate, polyoxyalkylene alkyl ethers, alcohol ethoxylates, polyethylene glycol nonylphenol ether, and organosilicon surfactants were all associated with increased activity levels for SsoPox (RA = 2.33–5.79) and GcL (RA = 1.00-1.17) (Table 1). Other ingredients appear to negatively affect the enzymatic activity levels, such as sodium salts of soya fatty acid, alkoxylated fatty acids and diethylene glycol, all found in Permeate (RA = 0.71 for GcL; RA = 1.04 for SsoPox) and whose individual contributions are not resolved. Lastly, we note that as highlighted above, all products containing non-ionic surfactants but also plant-based oils, such as Turbulance, Droplex, and Masterlock, result in low activity levels (Table 1).

The used polymers (pinene polymer, polyvinyl polymer, and polyacrylates), organic acids (amines, polyhydroxy-tricarboxylic acid) and salts (ammonium sulfate and dipotassium phosphate) showed overall neutral effects on the activity levels (RA = 0.67–1.36) of both enzymes, suggesting that they are well tolerated (Table 1). With petroleum products (petroleum oils, paraffin), we see that the activity of GcL is largely unaffected (RA = 0.95–1.07) while the activity of SsoPox is stimulated for two adjuvants (Peptoil and Lox; RA = 1.38–2.33) and reduced for a third (Herbi-oil; RA = 0.38) (Table 1).

These enzymes belong to two distinct protein families with SsoPox belonging to the phosphotriesterase-like lactonases with a characteristic (α/β)_8_ fold, and GcL belonging to the metallo-β-lactamase-like lactonases, adopting a αβ/βα fold^11^. There are no obvious biochemical differences between these two enzymes that could explain the differences in behavior seen here. Overall, this analysis suggests that GcL tolerates most chemicals within the tested adjuvants, with the exception of plant-based oils, and show very little activity variation (RA_min_ = 0.31; RA_max_ = 1.17). Conversely, the activity level of SsoPox varied more significantly (RA_min_ = 0.06; RA_max_ = 5.79) and the enzyme appeared largely inhibited by certain compounds such as plant-based oils, and highly stimulated by other chemicals, particularly surfactants.

### Formulation of lactonases in coatings and their characterization

**Fig. 3:**
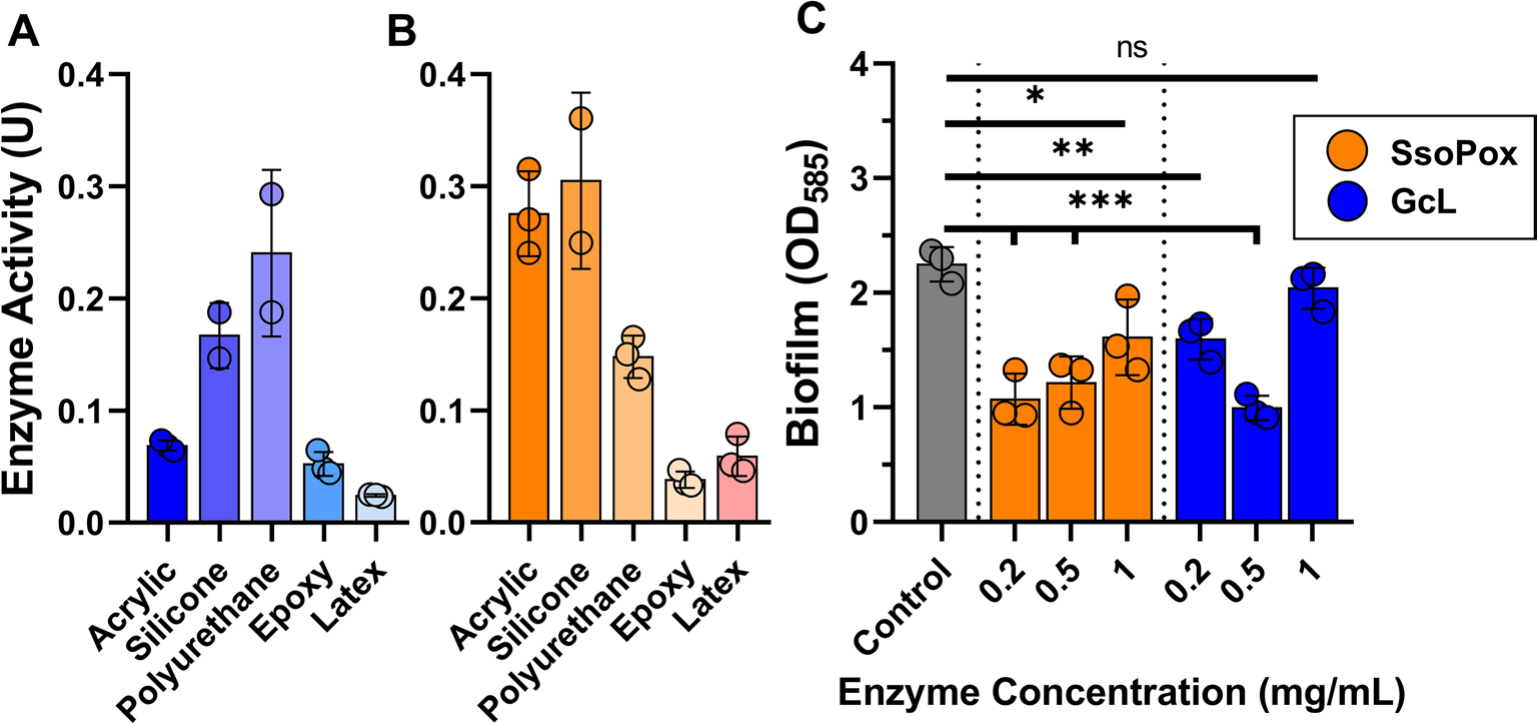
Tested lactonases are active in a variety of conditions including various coating chemistries, temperatures and salinity, and Acrylic coating with lactonase is shown to reduce biofilm. SsoPox (orange bars; **A**) and GcL (blue bars; **B**) (1 mg/mL) were combined with various coatings, and their paraoxonase activity was measured in acrylic (n = 3), silicone (n = 2), polyurethane (n = 3), epoxy (n = 3), and latex (n = 3) (see “Coating formulations and activity measurements” in methods). (**C**) SsoPox (orange) and GcL (blue) at concentrations between 0.2-5 mg/mL in an acrylic coating were tested for their biofilm reduction capabilities of *Pseudomonas aeruginosa* PAO1 using crystal violet (OD_585_ nm) (see “Biofilm Inhibition assay” in methods). Control indicates experiment with no enzyme treatment. Statistical significance was determined via a one-way ANOVA with a turkey multiple comparisons test. “ns” indicates *p *≥ 0.05, *indicates *p *< 0.05, **indicates *p* < 0.01, ***indicates *p* < 0.001.

Previous reports highlight that enzymes can be formulated into coatings^38,57^, including the lactonase SsoPox^15,44,45^. Here we investigate whether another lactonase, namely GcL, could be formulated into paint, and what is the compatibility of both lactonases with different coating bases. We diluted the lactonases into acrylic, epoxy, silicone, polyurethane, and latex marine paints, and evaluated the enzymatic activity of functionalized coatings via a chromogenic enzymatic assay (see methods; Fig. 3A and B).

Remarkably, both enzymes remained active in all tested coatings, yet measured activity levels vary widely in the different coatings. For instance, coating formulations with SsoPox showed highest enzymatic activity (reported as unit “U”, which is equivalent to 1 µM of substrate catalyzed per minute per mg of enzyme) with silicone (U = 0.31) and acrylic (U = 0.28) coating bases, while polyurethane (U = 0.15), epoxy (U = 0.04) and latex (U = 0.06) bases resulted in lower activity levels (Fig. 3A). GcL is most active in polyurethane (U = 0.24) and silicone (U = 0.17) bases, while acrylic (U = 0.07), epoxy (U = 0.05) and latex (U = 0.02) show lower activity levels (Fig. 3B). The three most favorable coatings are the same for both enzymes (acrylic, silicone and polyurethane), while the two least favorable ones are epoxy and latex. Possible explanations for the measured differences in activity between the coating formulations could relate to the difference in stability, activity levels, surface accessibility, or a combination of these parameters. Overall, the enzymes retain activity in all tested coating bases.

Quorum quenching lactonases such as SsoPox and GcL are known to reduce biofilm formation of certain bacteria^43^. We verified that a produced coating formulation in acrylic base can reduce biofilm formation using the model organism *Pseudomonas aeruginosa PAO1* (Fig. 3C**)**. The acrylic coating was chosen for this experiment as it is a versatile coating commonly used in many different kinds of ship hull antifouling coatings^58^. Both enzymatic formulations showed the lowest biofilm inhibition at the highest tested enzyme concentration of 1 mg/mL, with SsoPox having a ~ 30% reduction and GcL having a ~ 9% reduction in biofilm. At lower concentrations (0.5 and 0.2 mg/mL) SsoPox showed much greater biofilm inhibition at around ~ 50% each, whereas GcL showed some variation with a 55% biofilm reduction at 0.5 mg/mL, and a 29% biofilm reduction at 0.2 mg/mL. This is consistent with previous results of dose-dependent lactonase biofilm inhibition, where the highest concentration of SsoPox and GcL tested were not the most effective at biofilm inhibition^49^. While this experiment has limitations, e.g. bacterial cell density of the used culture is high and it relates to a single species biofilm, it is consistent with the previously observed ability of the SsoPox enzymatic coating to reduce biofouling and affect surface microbial communities^45^.

**Fig. 4:**
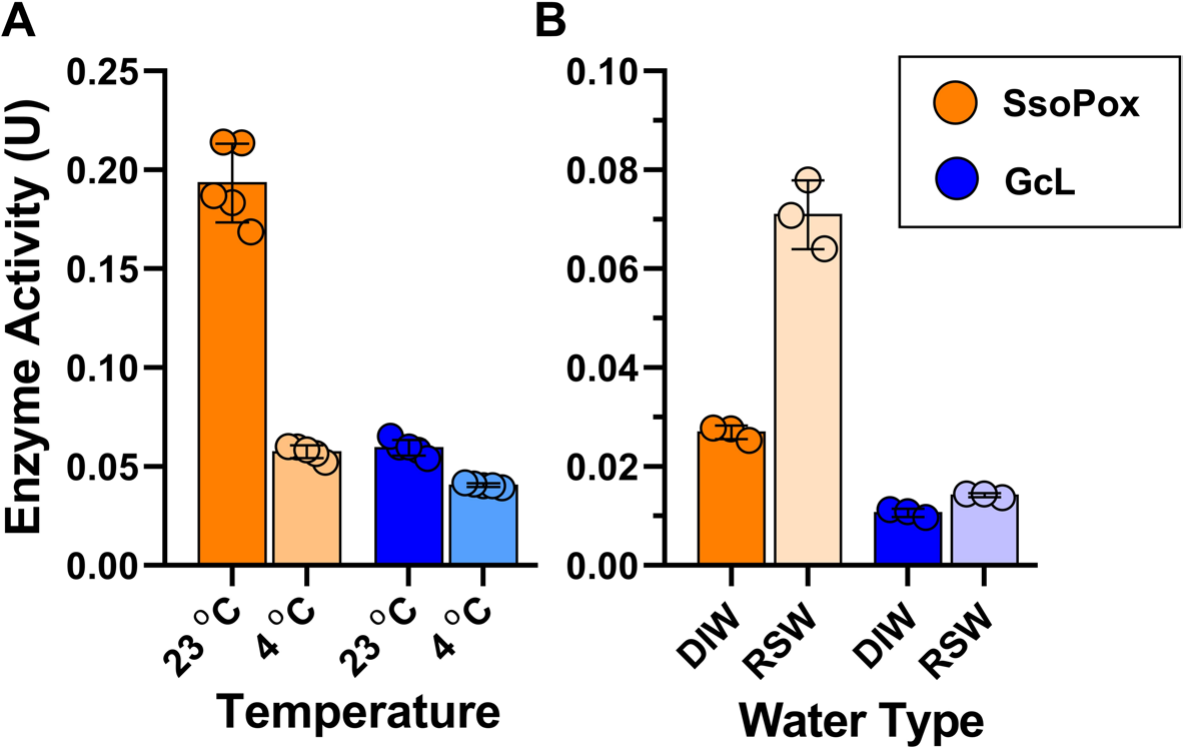
Formulated acrylic lactonase coating activity differs with temperature and salinity. (**A**) SsoPox (orange bars) and GcL (blue bars) at 1 mg/mL were tested at different temperatures (23 ^o^C and 4 ^o^C) (see “Coating formulations and activity measurements” and “Evaluation of coating activity in different conditions” in methods) (**B**) SsoPox (orange bars) and GcL (blue bars) at 1 mg/mL in an acrylic coating were tested for activity in deionized water (DIW), and reconstituted seawater (RSW). Enzymatic activity is measured with ethyl-paraoxon substrate. Enzyme activity is reported as U, which is 1 µM of substrate catalyzed per minute.

We also evaluated the effects of some physicochemical properties (salinity and temperature) on the activity level of the functionalized acrylic coating. Because enzymes are catalysts, rates are expected to decrease at lower temperatures^59^. Here, we compared the activity levels at two temperatures, 23 °C and 4 °C (Fig. 4A). These two temperatures are representative of water temperatures in different climates. For the acrylic coating functionalized with SsoPox, the activity level dropped ~ 3-fold between the two temperatures (0.19 U at 23 °C, 0.06 U at 4 °C), whereas GcL-containing coating saw a much more modest activity decrease at low temperature (0.06 U at 23 °C, 0.04 U at 4 °C). We examined the effect of high salinity on the coating enzymatic activity. We compared the activity levels in deionized water (DIW) and in reconstituted seawater (RSW; Fig. 4B). For both enzymatic coating formulations, activity levels are increased in RSW compared to DIW. Indeed, the activity level is ~ 2.6-fold higher for SsoPox (0.027 U in DIW, 0.07 U in RSW) and ~ 1.3-fold higher for GcL (0.011 U in DIW, 0.014 U in RSW).

### Evaluating and reducing enzyme leakage

Leakage of active molecules from paint can be a significant issue^38^. In the case of biocides, their release can however be an effective barrier to fouling, and numerous commercial coatings known as ablative are designed to leak biocides^60^. With lactonase enzymes, leaking molecules is expected to result in lower material functional activity.

**Fig. 5:**
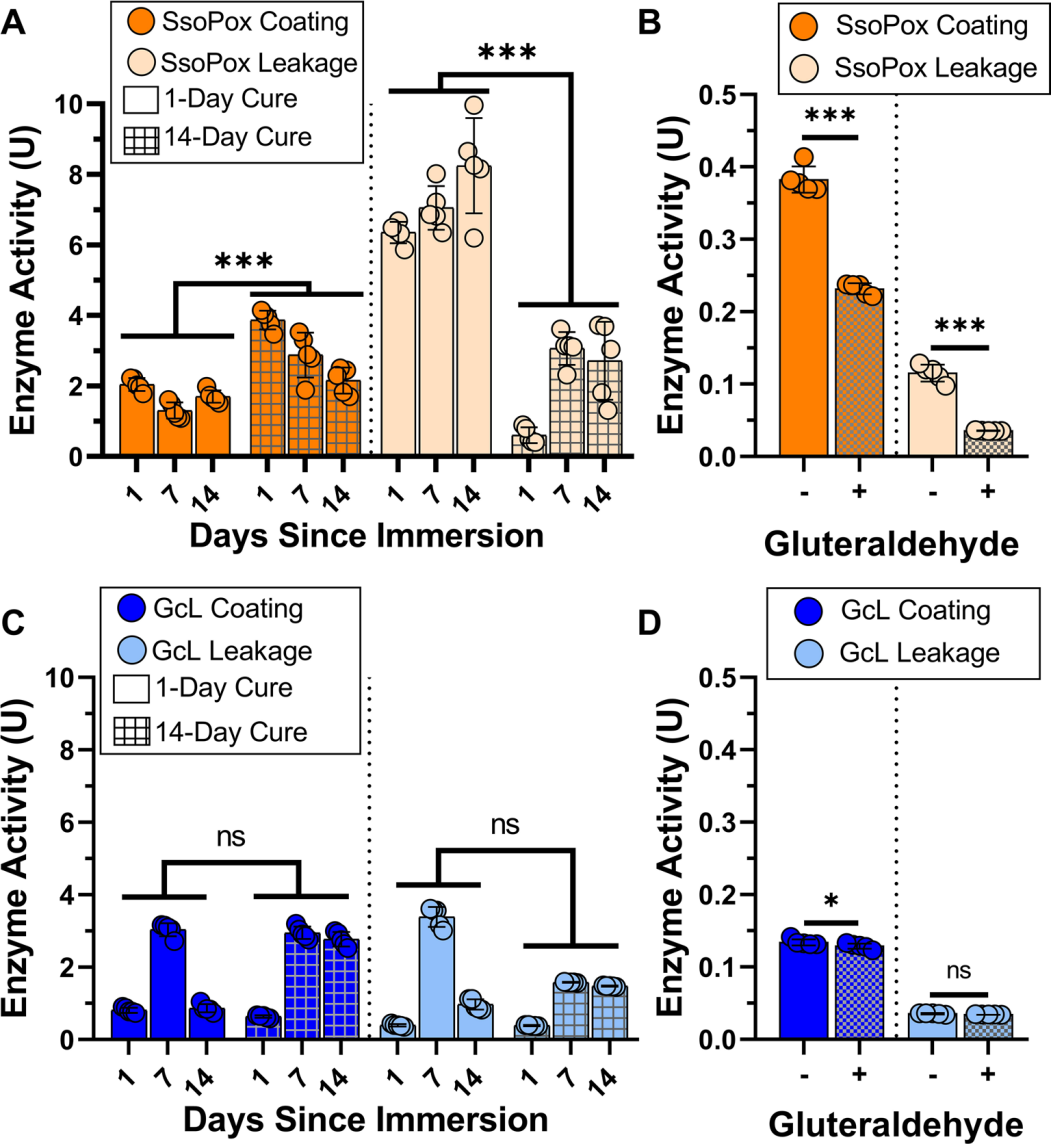
Evaluation of enzymatic activity in acrylic coating as a function of cure time and addition of the crosslinker glutaraldehyde. Effect of cure time on Ssopox (**A**) and GcL (**C**) formulations activity levels (in coating) and on enzyme leakage (in surrounding liquid) (see “Coating formulations and activity measurements” and “Glutaraldehyde enzyme immobilization” in methods). Statistical significance was determined via a two-way ANOVA using a Geisser-Greenhouse correction with a Turkey multiple comparison. “ns” indicates *p ≥* 0.05, ***indicates *p* < 0.001. Effect of glutaraldehyde (3%) on coating and leakage enzymatic activity for SsoPox (**B**) and GcL (**D**). Statistical significance was determined via a one-way ANOVA. “ns” indicates *p ≥* 0.05, ***indicates *p* < 0.001. Paraoxon was used as substrate.

Here, we evaluated the importance of cure time as well as chemical crosslinking using glutaraldehyde for the acrylic base enzymatic formulation. We used two curing times, 1-day and 14-day cure. After a 1-day cure time, the activity of the SsoPox acrylic coating was 2.04 U after 1 day of submersion, 1.30 U after 7 days of submersion, and 1.70 U after 14 days of submersion (Fig. 5A). Significant leakage occurred, with high activity levels that leaked from the coating to the water (6.35 U, 7.05 U and 8.24 U, after 1, 7 and 14 days of submersion). We note that while the activity levels in water are higher than measured activity in the coating, this does not necessarily mean that most of the enzyme leaked out. Indeed, because enzyme was diluted in the paint, most of enzyme molecules are likely inaccessible to the substrate and trapped in the coating polymer, resulting in lower measured activity levels. This makes it so the enzyme activities of the coating and leaked enzyme cannot be directly compared. With the 14-day cure time, the coating activity increased significantly (~ 1.75-fold on average, e.g. 3.87 U versus 2.04 U after 1 day of submersion) (Fig. 5A). Activity levels decreased over time with submersion (3.86 U, 2.88 U and 2.16 U, after 1, 7 and 14 days of submersion). Enzyme leakage also significantly decreased compared to 1-day cure results (~ 4-fold on average; 0.60 U versus 6.35 U after 1 day of submersion). Leaked activity increased to 3.06 U and 2.71 U after 7 and 14 days of submersion, respectively.

With GcL containing formulations, no differences were observed with different curing times (Fig. 5C). Over the course of the 14 days of submersion, activity levels in the coating ranged from 0.81 to 3.03 U after 1-day cure, and 0.64–2.95 U after 14-day cure (Fig. 5C). Similarly, leaked enzyme levels were nearly unchanged at low levels (Fig. 5C.40–3.38 U after 1-day cure, and 0.39–1.58 U after 14-day cure).

We also evaluated the ability of glutaraldehyde treatment to reduce enzyme leakage. Glutaraldehyde is a well-studied enzyme fixative that is used to bind enzymes to solid matrices^61,62^. Addition of glutaraldehyde resulted in both reduced activity of the enzyme coating as well as reduced leakage (Fig. 5B and D). For SsoPox acrylic coating, glutaraldehyde reduced the activity of the coating by ~ 62%, whereas it also reduced the leaked enzyme activity by a greater margin with an ~ 82% reduction in leakage activity over the 1-day testing period (Fig. 5B). GcL showed more modest changes with the addition of glutaraldehyde with a ~ 45% reduction in activity in the coating and a ~ 26% reduction in leakage in the 1 day tested (Fig. 5D).

### Evaluation of enzymatic coating longevity

A major challenge of using enzymes for industrial purposes is their lack of stability over time^63^. We evaluated the activity of enzymatic acrylic coating over time in a 96-well plate format, in which well bottoms were coated. We evaluated the enzymatic acrylic coatings with SsoPox and GcL in dry and wet (deionized water) conditions over time. Here we used a lactone substrate (TBBL)^47^, a close mimic to the signals acyl homoserine lactones and that allows for easy measurement, with color change (see Methods).

**Fig. 6:**
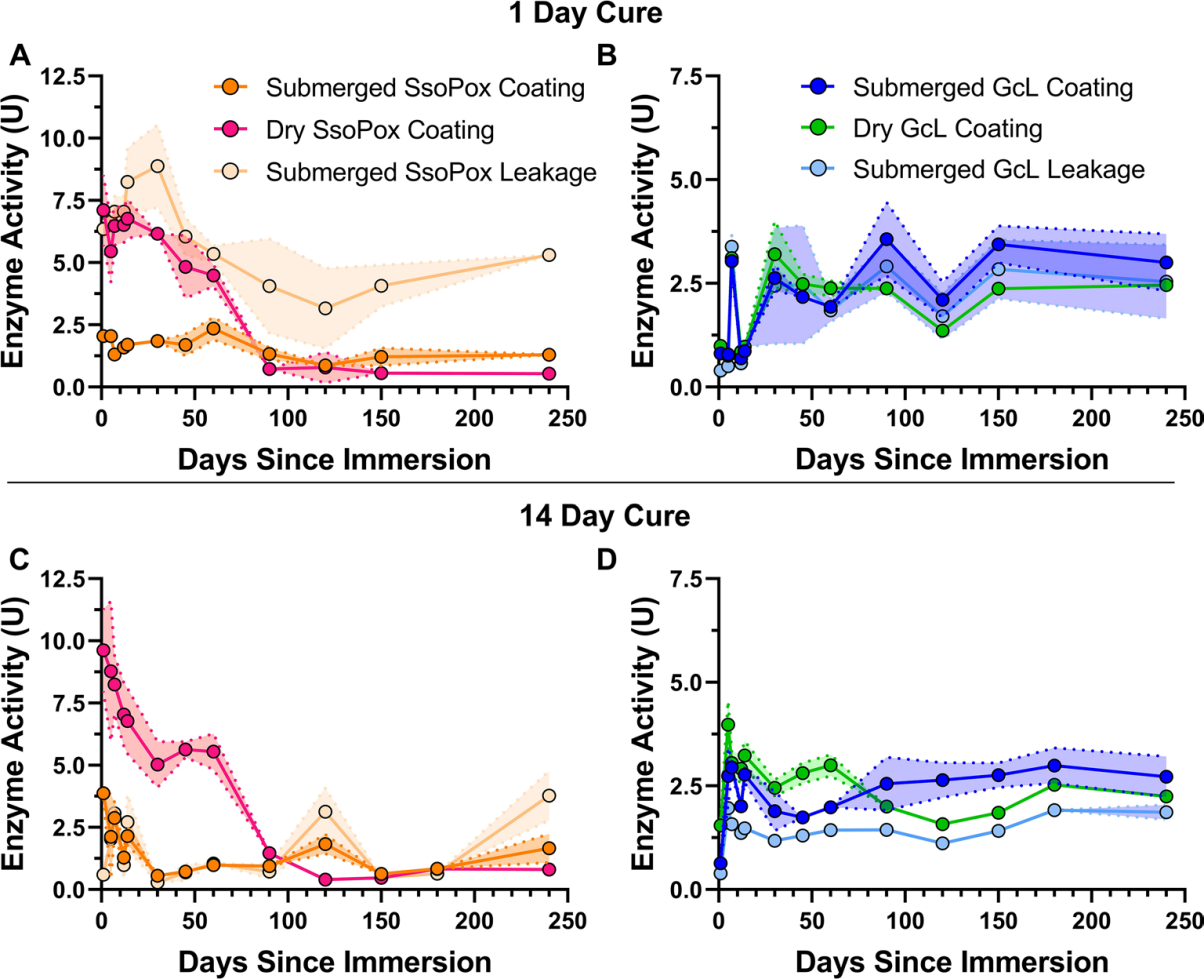
Enzymatic acrylic coating longevity. Coated samples (*n* = 5 for each time point) with SsoPox (**A**, **C**) and GcL (**B**, **D**) were stored in dry conditions or submerged in DIW (see “Long term coating durability experiment” in methods). Lactonase activity is measured with TBBL substrate. Standard deviation is shown by the highlighted area around the line. Coatings were either cured for 1 day (**A**, **B**) or 14 days (**C**, **D**).

Results show that both enzymatic coatings remain active over a 250-day period when submerged in water (Fig. 6). In fact, the SsoPox coating retained > 60% of its initial activity (Fig. 6A; initial activity = 2.0 U, activity at day 250 = 1.3 U). The formulation with GcL showed increased activity over time when submerged (Fig. 6B; initial activity = 0.81 U, activity at day 250 = 3.0 U). A possible cause for this includes coating structural changes that make more enzyme accessible to degrade the substrate. SsoPox enzyme leakage was significant, although its level did not substantially increase over the time course of the experiment (Fig. 6A; initial activity = 6.35 U, activity at day 250 = 5.3 U). Similarly, with GcL formulation, enzyme leakage remained at a stable level (Fig. 6B; initial activity = 0.4 U, activity at day 250 = 2.5 U).

When stored in dry condition, the formulation with SsoPox showed significant decrease over time (Fig. 6A; initial activity = 7.1 U, activity at day 250 = 0.4 U), suggesting that substantial level of activity was lost over time, within the first three months of the experiment (day 90 = 0.7 U). The formulation with GcL behaved quite differently, since activity levels in dry condition showed an increase over time, similarly to its behavior in wet condition (Fig. 6B; initial activity = 1.0 U, activity at day 250 = 2.5 U).

There was very little change in coating performance when cure time was extended to 14 days (Fig. 6C and D), with the only notable change being a significant decrease in SsoPox leakage of the submerged paint as the average enzyme leakage over the course of the experiment for the 1-day cure was ~ 6 U, and this was decreased by a factor of ~ 4 to ~ 1.5 U for the 14-day cure.

## Conclusion

This study provides a comprehensive evaluation of formulation strategies for two thermostable quorum quenching lactonases with potential applications in microbial control, both to mitigate crop disease and inhibit marine biofouling. We investigated two different approaches: incorporation with adjuvants for solution/spray applications and integration into polymer matrices for functionalized coatings. Previous work showed that quorum quenching lactonases can reduce crop disease and biofouling, yet the functionalization and the durability of enzyme formulations are largely underexplored. Both enzymes showed remarkable compatibility with most tested surfactants, indicating their potential as enzymatic additives to existing products. When incorporated in coating polymers, the two lactonases maintained activity across all five tested coating bases although substantial variation in activity levels occurred depending on the polymer type. Notably, the functionalized acrylic coating exhibited exceptional stability, maintaining activity levels over the tested time frame (250 days), under both wet and dry conditions. Our findings unexpectedly reveal that GcL, despite its lower thermostability (T_m_ = 67.82 °C compared to 87.8 °C for Ssopox), demonstrated comparable stability in the tested polymers and chemicals, suggesting that moderately thermostable enzymes could be sufficient for harsh conditions. This work not only confirms the biotechnological potential of thermostable lactonases for industrial applications, but also establishes practical formulation strategies. These results provide a foundation for future field evaluations of enzymatic solutions for bacterial control in real-world applications.

## Electronic supplementary material

Below is the link to the electronic supplementary material.

Supplementary Material 1

## Data Availability

The data for this study is available at the Open Science Foundation (DOI 10.17605/OSF.IO/KJBY8).
