# Supplementary material for: Durable formulations of quorum quenching enzymes
Source: Sci Rep. 2025 Jul 28;15:27435. doi: 10.1038/s41598-025-12623-1 (PMC12304125; doi:10.1038/s41598-025-12623-1)
Supplement: Supplementary file 1 — Supplementary Material 1 [file 41598_2025_12623_MOESM1_ESM.pdf]

Supplementary material for

Durable Formulations of Quorum Quenching Enzymes

Reed Jacobson[1†], Colton Castonguay[2†], Mikael H. Elias[1,2#]

[1]University of Minnesota, Department of Biochemistry, Molecular Biology and Biophysics, St. Paul, MN, 55108, USA.

[2]Biotechnology Institute, St. Paul, MN, 55108, USA.

[†] Contributed equally

[#]Correspondence:

Tel: 1-612-626-1915; E-mail: mhelias@umn.edu

Table of content

Table S1 Adjuvant classification and active ingredients.. 3

Table S2 Adjuvant manufacturer's information.. 4

Figure S1. Melting curve of lactonase enzyme GcL... 5

Classifications	Product Name	Active Ingredients (as listed by manufacturer)
**De-foaming agent**	AMS Xtra	Ammonium sulfate
**Oils**	Droplex	Modified vegetable oil, polyoxyethylene sorbitan fatty acid ester, vegetable oil
	Turbulance	Methylated Vegetable oil, polyether modified polyisiloxane, alkyphenol ethyoxylte
	MES 100	Alkylphenol ethoxylate, methylated seed oil
	Pinene II	Methylated Seed Oil, alcohol ethoxylates, pinene (diterpene) polymer
	Masterlock	Modified vegetable oil, polyoxyethylene sorbitan fatty acid ester, vegetable oil, ethoxylated soibean oil
	Peptoil	Petroleum oil, paraffin base, alkylphenol ethoxylate, alcohol ethoxylate
	Herbi-oil	Parrafin based petrolium hydrocarbons, oil-soluble surfactant blend
**Surfactants**	Surf-Ac 910	Polyethylene glycol nonylphenyl ether, alcohol ethoxylates
	Sil-Fact	Alcohol ethoxylates, organosilicone surfactant
	Permeate	Alkoxylated fatty acid, sodium salts of soya fatty acid, diethylene glycol
	Wick	Polyoxyalkylene alkyl ethers
**Deposition Aids**	LOX	Amine salts of organic acid, organic acid, aliphatic petroleum distillate, aromatic petroleum distillate
	Clasp	Polyvinyl polymer
**Water Conditioner**	U-Surp	Proprietary blend of polyacrylates, polyhydroxy-tricarboxylic acid, dipotassium phosphate and formulation aids
**Sticking Agent**	Transfix	Beta pinene polymer

Table S1 Adjuvant classification and active ingredients. Adjuvants were classified as oils if they contained an oil-based compound as their first active ingredient, otherwise they were classified according to manufacture advertisements on their website. Adjuvant active ingredients were found on adjuvant manufacturer website or were listed by the manufacturer on the container.

Manufacturer	Product Name	Ratio	Concentration
Helena Chemical Company	Clasp	2.5 µl / 1 ml	0.25%
	Pinene 2	1 µl / 2.134 ml	0.04%
	Mes-100	1.25 µl / 1 ml	0.13%
	Surf-AC 910	1.25 µl / 1 ml	0.13%
Drexel Chemical Company	U-Surp	1 µl / 2.134 ml	0.04%
	AMS-ALL	10 µl / 1 ml	1.00%
	LOX	1 µl / 2.134 ml	0.04%
	Peptoil	10 µl / 1 ml	1.00%
	Sil-Fact	1 µl / 2.134 ml	0.04%
Winfield United	Herbi-Oil	10 µl / 1 ml	1.00%
	Wick	1.812 µl / 1 ml	0.18%
	Droplex	1.56 µl / 1 ml	0.16%
	Turbulence	1.25 µl / 1 ml	0.13%
	Permeate	1.25 µl / 1 ml	0.13%
	Transfix	1 µl / 2.134 ml	0.04%
	Masterlock	1 µl / 2.134 ml	0.04%

Table S2 adjuvant manufacturer’s information, product name, and recommended ratio (values converted to µL/mL), and respective % concentration

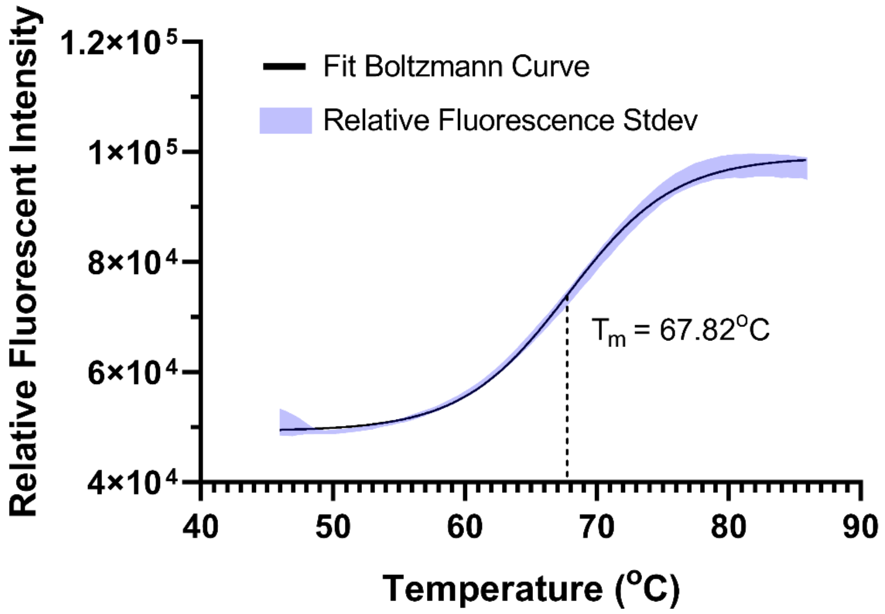

Figure S1. Melting curve of lactonase enzyme GcL.

GcL was combined with SYPRO orange, then heated in a thermocycler while fluorescence was measured. The data was fit with a Boltzmann sigmoidal curve (black line; $r^2 = 0.995$). The standard deviation of the measured relative fluorescence is shown in blue. The melting temperature (T_m) is at the curve's V50, which here is 67.82 °C.

Methods: GcL Enzyme was purified as previously described (Bergonzi et al., 2019). 10 µL of 5 µM GcL was combined with 5 µL of 200X SYPRO orange and 35µL PTE buffer, and mixed in an appliedbiosystems MicroAmp Fast 96-well Reaction Plate in triplicate. The plate was then sealed with applied biosystems Optical Adhesive Cover, then placed in an applied biosystems StepOnePlus thermocycler. Samples were run for ~2 hours starting at 25 °C and ending at 95 °C, taking measurements approximately every minute. Fluorescence was measured, then plotted with a Boltzmann sigmoidal curve, with the curve's V50 as the protein's melting temperature. Data points outside the 45 °C-85 °C range were excluded for analysis to capture the sigmoidal portion of the curve.

Reference

Bergonzi, C., Schwab, M., Naik, T., & Elias, M. (2019). *The Structural Determinants Accounting for the Broad Substrate Specificity of the Quorum Quenching Lactonase GcL*. 1–9. https://doi.org/10.1002/cbic.201900024
